# Genetic Susceptibility to Sport-Related Muscle Injuries: Insights from the Literature and Novel Gene Candidates

**DOI:** 10.3390/ijms262211175

**Published:** 2025-11-19

**Authors:** Agata Leońska-Duniec

**Affiliations:** Faculty of Physical Education, Gdansk University of Physical Education and Sport, 80-336 Gdansk, Poland; agata.leonska-duniec@awf.gda.pl

**Keywords:** sports genetics, genetic susceptibility, polymorphisms, injury risk, injury prevention, skeletal muscles, physical activity

## Abstract

Physical activity is widely recognized for its health benefits; however, it also increases the risk of musculoskeletal soft tissue injuries, with muscle-related cases constituting a considerable proportion. These injuries impair well-being, athletic performance, and career longevity while creating substantial social and economic burdens. Their multifactorial etiology involves internal and external risk factors, and evidence suggests a heritable component influencing tissue integrity, recovery, and overall susceptibility. While genetic contributions to ligament and tendon damage are relatively established, knowledge regarding muscle injuries remains limited. This review critically summarizes current evidence on polymorphisms associated with sport-related muscle injury susceptibility. A systematic search of PubMed, Scopus, and Web of Science identified studies examining genetic markers in physically active individuals with documented muscle injuries. To date, 37 single nucleotide polymorphisms in 32 genes have been significantly linked to injury risk, recurrence, severity, and recovery. These genes cluster into categories involving muscle structure, growth and regeneration, metabolism, inflammatory and stress responses, membrane stability, signaling, and vascular regulation. By integrating available findings and outlining knowledge gaps, this review highlights promising directions for advancing personalized prevention and rehabilitation strategies in sports medicine.

## 1. Introduction

Although physical activity offers numerous health benefits, it is also associated with an increased risk of musculoskeletal soft tissue injuries, 66.6% of which are caused by noncontact mechanisms. Muscle injuries account for approximately 25% of all sport-related injuries and tend to recur in 16% of patients [1]. They have notable implications at multiple levels. For individuals, such injuries often result in pain, reduced mobility, and prolonged recovery, affecting not only physical health but also mental well-being and quality of life. For professional athletes, they may interrupt careers, reduce performance, or lead to early retirement, which also affects teams and the sports industry. On a social level, these injuries increase the demand for health care services and rehabilitation and may discourage physical activity in the general population. They also generate both direct costs, such as medical treatment and rehabilitation, and indirect losses, including absenteeism and reduced productivity [2]. For instance, a study of Spanish professional football clubs revealed that the high number of muscle injuries can cost approximately €365,811 per team each month [3]. Additionally, the broader economic burden of physical inactivity has been estimated at 0.3–4.6% of national health care expenditures [4]. These impacts highlight the need for effective prevention, early intervention, and targeted rehabilitation strategies, especially in high-risk groups.

Muscle injuries are difficult to define and characterize because of their multifactorial nature [5]. Numerous risk factors have been suggested, which can be stratified into internal and external components. The former are related to age, origin, sex, and previous soft tissue injuries. The latter include altered muscle length, decreased flexibility, strength imbalance, insufficient warm-up, exercise volume, and fatigue [6]. Additionally, familial and case–control association studies have provided evidence that susceptibility to muscle injury has a heritable, genetic component. Inherited factors may contribute to interindividual differences in response to mechanical load and thus may influence tissue integrity, function, and ultimately musculoskeletal injury susceptibility. This heritability has been shown to be polygenic in nature rather than being due to a single genetic variant [7]. Genetic predispositions to an increased risk of anterior cruciate ligament (ACL) injuries are relatively well documented [8]. However, the genetic factors influencing susceptibility to muscle injury have only recently begun to attract scientific attention and remain largely unexplored.

The aim of this review is to critically evaluate the current knowledge on genetic factors contributing to susceptibility to sport-related muscle injuries, identify consistent findings across previous studies, and propose novel candidate genes for future research based on biological plausibility and recent advances in genomics. For this purpose, a literature review was performed to identify all existing records of studies on muscle injuries and genetic markers. Three databases, namely, PubMed, Scopus, and Web of Science, were searched using the following search terms: “polymorphism” OR “gene” OR “SNP” OR “genetic variation” AND “injury muscle” AND “sport” OR “physical activity”. The inclusion criteria for articles were as follows: (i) original research studies, (ii) written in English, (iii) published in a peer-reviewed journal, (iv) involved human, (v) participants with diagnosed or reported muscle injuries, and (vi) physically active participants. Studies concerning individuals with additional disease, reviews, books, book sections, opinion articles, letters to the editor, film/broadcasts, theses, and congress abstracts were excluded.

## 2. Genes Significantly Associated with Susceptibility to Sport-Related Muscle Injuries

To date, only 37 single nucleotide polymorphisms (SNPs) localized in 32 genes have been significantly associated with muscle injury risk, incidence, recovery time, and/or severity (Table 1). The products of these genes can be divided into six main functional categories: (i) structural components of muscle fibers and the extracellular matrix (ECM); (ii) factors influencing muscle growth, development, and regeneration; (iii) regulators of metabolism and energy homeostasis; (iv) inflammatory and stress-response factors; (v) cell membrane stability, ion channels, and signaling influences; (vi) regulators of vascular function and perfusion.

### 2.1. Genes Involved in the Structural Organization of Muscle Fibers and ECM

Genes encoding structural elements of muscle tissue, such as actin, myosin, titin, collagen, or EMC, can significantly affect sarcomere integrity and flexibility, muscle contraction, and force transmission, which clearly identifies them as candidate genes. These genetic variations may lead to changes in muscle fiber composition, elasticity, tensile strength, or the ability to repair damage. As a result, individuals carrying certain genotypes may be more prone to muscle injuries, especially during intense or prolonged physical activity. The genes examined include those that produce the structural elements of sarcomeres, e.g., *ACTN3*, and EMC, e.g., collagen and metalloproteinase genes, *DCN*, and *ELN* (Table 1) [34,35].

The best-studied gene in this context is *ACTN3*, which encodes α-actinin-3, an important structural component of the sarcomere Z-disk in fast-twitch (type II) muscle fibers, where it anchors actin filaments. A common polymorphism in this gene, known as R577X (rs1815739), results in a transition of cytosine (C) to thymine (T), which leads to a stop codon (allele X) instead of the arginine codon (allele R) at position 577 of the protein sequence. Consequently, the XX genotype results in complete sarcomeric α-actinin-3 deficiency [36,37]. Although the absence of α-actinin-3 is not associated with any specific disease or clinical disorder, research has indicated that it may lead to several less favorable physical traits. These include decreased muscle strength, smaller muscle size, lower tolerance to high-intensity muscular exertion, and, in some cases, reduced bone mineral density [38,39]. Numerous studies have reported that the RR genotype tends to be more prevalent among elite athletes specializing in speed- and power-based disciplines [40]. Conversely, the nonfunctional XX genotype has been found at greater frequencies among certain elite endurance athletes [41]. In addition, this protein deficiency may disrupt the structural integrity and resilience of muscle fibers under mechanical stress, thereby increasing the risk of muscle damage or injury during high-intensity exercise. A systematic review of 13 studies involving more than 1000 athletes, mainly football players, across six countries revealed that the XX genotype is consistently associated with an increased incidence of noncontact muscle injuries in 12 of those investigations [42]. In addition, more severe injuries and longer recovery times have been identified in players carrying at least one X allele than in those with the RR genotype [15]. However, some studies have reported no associations between any *ACTN3* genotypes and muscle injury risk in elite endurance runners or university athletes [12,14,40,41,43,44]. At least one study has also suggested that individuals with the RR or RX genotype may face an increased risk of muscle injury during participation in various sports [45]. Moreover, carriers of the R allele have been shown to exhibit greater passive stiffness in the hamstring muscles than those with the XX genotype; however, these altered mechanical properties might not affect the risk of hamstring muscle strain injury [43]. This evidence has shown that the link between the X allele and sport-related muscle injuries is not universal, especially in endurance or resistance training contexts, highlighting the importance of considering sport type, the environment, and a polygenic background for accurate injury risk prediction and personalized prevention.

Collagen genes encode various types of collagen proteins, which are key structural components of connective tissues, including tendons, ligaments, and ECM surrounding muscle fibers. These proteins contribute to the strength, flexibility, and integrity of the musculoskeletal system. Variations in collagen-related genes can influence the biomechanical properties of these tissues, potentially affecting an individual’s susceptibility to soft tissue injury during physical activity. The most studied collagen genes in the context of muscle injury include *COL1A1*, *COL5A1*, and *COL22A1* [12,20,21,22,23,24,46].

In particular, the *COL5A1* gene, which encodes the α1 chain of collagen type V, plays a critical regulatory role in collagen fibrillogenesis, controlling the diameter and organization of collagen fibers, which form part of ECM of skeletal muscles [47]. A well-characterized polymorphism in this gene, rs12722 (C/T), has been associated with differences in connective tissue properties. Individuals with the TT genotype have been reported to exhibit stiffer and less extensible connective tissues, which may reduce the ability of muscle-tendon units to effectively absorb and distribute mechanical loads. As a result, these individuals may be at greater risk for muscle strains, tears, and other soft tissue injuries during high-intensity or repetitive physical activity [48]. In one study, Massidda et al. (2015) reported that this genetic variant could explain up to 44% of the variation in injury severity, suggesting a relationship with the TT genotype [46]. In a study by Pruna et al. [22], which was also conducted on football players, a significant association was identified between the TC genotype and more severe injuries. In addition, several studies have shown a link between the *COL5A1* rs12722 polymorphism and an increased incidence of exercise-induced muscle damage, particularly in sports involving endurance or explosive movements. This variant is believed to influence collagen turnover and tissue repair capacity, making it a potential genetic marker for identifying athletes with elevated injury risk. For example, Baumert et al. [35] reported that individuals carrying the T allele of the rs12722 SNP reported higher levels of muscle soreness following eccentric exercise, suggesting an altered collagen remodeling response. Furthermore, a meta-analysis by Pabalan et al. [49] confirmed that the TT genotype is associated with an increased risk of tendon and ligament injuries, supporting the role of *COL5A1* in connective tissue integrity and recovery.

### 2.2. Genes Related to Muscle Growth, Development, and Regeneration

The second important group of genes encodes factors that influence muscle growth, development, and regeneration. Key representatives of this group include insulin-like growth factor 1/2 (*IGF1*/*2*), myostatin (*MSTN*), myogenic differentiation 1 (*MYOD1*), myogenin (*MYOG*), myogenic factor 4/5 (*MYF4/5*), and paired box 3/7 (*PAX3/7*) genes. They are involved in various biological pathways that regulate muscle hypertrophy, protein synthesis and degradation, inflammation, and cellular repair mechanisms. Variations in genes involved in these processes may influence how individuals respond to training and how effectively they repair muscle tissue after injury [50].

In skeletal muscle, IGF2 contributes to myogenesis by stimulating the proliferation of myoblasts and promoting their differentiation into mature muscle fibers. Although its expression decreases after birth, IGF2 continues to influence muscle regeneration in adulthood by supporting satellite cell activation and modulating protein synthesis pathways. Variants in the *IGF2* gene have been associated with differences in muscle mass, strength, and recovery capacity, suggesting that its genetic variability may contribute to interindividual susceptibility to muscle injury and the efficiency of postinjury repair [51]. The relationship between the rs3213221 *IGF2* polymorphism and muscle injury was repeatedly shown by Pruna and coworkers in three studies. First, they reported that individuals with the GC genotype experienced less severe injuries than those with the homozygous GG or CC genotypes did [21]. Second, muscle injuries were related to the presence or absence of the G allele, whereas tendon injuries were related to the presence or absence of the C allele [25]. Third, compared with the CC or GG genotypes, the GC genotype was significantly associated with less severe injuries [22]. This relationship explains the important role of IGFs in soft tissue growth and the increased expression of these genes in response to degeneration and regeneration following injury. During this process, IGFs interact with other key genes, such as fibroblastic growth factor (*FGF*), interleukin 1 β (*IL1B*), *IL6*, and transforming growth factor-β (*TNFβ*), to support satellite cell activation and muscle repair [52].

### 2.3. Genes Associated with Metabolism and Energy Homeostasis

Genes associated with metabolism and energy homeostasis play pivotal roles in determining muscle performance and susceptibility to injury. Variants in genes, such as *AMPD1*, peroxisome proliferator-activated receptor gamma coactivator 1 α/β (*PPARGC1A/B*), and *CKM*, affect biochemical pathways that regulate energy availability, mitochondrial biogenesis, creatine metabolism, and oxidative capacity. By modulating recovery efficiency and muscular resilience to mechanical stress, these variants may indirectly influence susceptibility to muscle injury [50].

Polymorphisms in the *AMPD1* gene, which encodes adenosine monophosphate deaminase isoform 1, have been extensively studied because of their impact on muscle energy metabolism in muscle fibers, which shifts the equilibrium of the reaction of the purine nucleotide cycle towards adenosine triphosphate production through the conversion of adenosine monophosphate into inosine monophosphate [53]. The common rs17602729 (C34T) polymorphism in *AMPD1* introduces a premature stop codon at exon 2, resulting in the production of a truncated and nonfunctional protein. Previous research has shown that rs17602729 T allele carriers have diminished exercise capacity and cardiorespiratory responses to exercise, a limited training response of ventilatory phenotypes during maximal exercise, and a reduced submaximal aerobic capacity [53,54,55]. These changes result in impaired adenosine triphosphate (ATP) regeneration and early fatigue and prevent muscle cells from accessing the energy they need to function, leading to their death, which causes musculoskeletal injury. This finding was repeatedly shown by Maestro et al. [26], who reported that the *AMPD1* gene is a relevant factor for muscle injuries in professional soccer players. The TT genotype was associated with a greater risk of musculoskeletal injury, whereas the CC genotype had a protective effect. Varillas-Delgado et al. (2024) reported that the TT genotype is associated with an increased risk of sports injuries and emphasized the role of the *AMPD1* gene in injury risk in endurance athletes, suggesting that optimal muscle metabolism has an influence on injuries [56].

### 2.4. Genes Involved in Inflammatory and Stress-Response Pathways

Another important group of genes associated with susceptibility to muscle injury consists of those that regulate inflammatory signaling and cellular stress responses. Variants in genes such as *IL6*, *IL1B*, and *TNF* can influence the magnitude and duration of postexercise inflammation, oxidative stress, and catabolic activity, thereby affecting recovery efficiency and the risk of soft tissue injury under repeated or intense loading. A transient increase in proinflammatory cytokines following muscle damage supports repair and regeneration via myogenesis, but excessive or chronic activation of these pathways reduces regenerative capacity, promotes atrophy, and ultimately impairs tissue recovery [57].

The best-studied genetic marker in this group is the *IL6* rs1800795 polymorphism. IL-6 is secreted by skeletal muscle in response to exercise and exerts autocrine effects. Interestingly, it plays a dual role, functioning as a mediator of hypertrophic growth and myogenesis while simultaneously contributing to muscle atrophy and catabolism [58]. Larruskain et al. [12] reported that compared with the GC and CC genotypes, GG homozygotes are associated with an increased risk of hamstring injury. The G allele has been shown to increase *IL6* gene transcription and increase circulating IL-6 concentrations under stress conditions [59]. In contrast, healthy individuals carrying the CC genotype exhibit higher postexercise creatine kinase (CK) levels following eccentric contractions [60]. Additionally, in a study of 402 Caucasian male soccer players, Hall et al. [33] reported that CC homozygotes were more likely to experience any injury or a muscle injury than G allele carriers. They proposed the C allele as a risk allele for muscle damage and inflammation. These conflicting findings emphasize the context-dependent role of *IL6* variants and point to the importance of future studies aimed at clarifying their contribution, along with other genetic determinants, to individual susceptibility to muscle injury.

### 2.5. Genes Associated with Membrane Integrity, Ion Transport, and Signaling Pathways

Genes responsible for membrane stability and signaling, e.g., anoctamin 5 (*ANO5*), caveolin 3 (*CAV3*), dysferlin (*DYSF*), and the sarcoglycan genes, as well as those regulating ion channel function, e.g., ryanodine receptor 1 (*RYR1*), ATPase sarcoplasmic/endoplasmic reticulum Ca^2+^ transporting 1 (*ATP2A1*), calcium voltage-gated channel subunit α1 S (*CACNA1S*), sodium voltage-gated channel α subunit 4 (*SCN4A*), and chloride voltage-gated channel 1 (*CLCN1*), are essential for maintaining excitation–contraction coupling, calcium homeostasis, and resistance to fatigue. Variants within these genes may compromise sarcolemmal integrity, alter ion flux, and impair recovery from mechanical stress, thereby potentially influencing susceptibility to exercise-induced damage and athletic injury risk [61,62].

One of the candidate genes is *CASP8*, which encodes a protease that initiates the extrinsic apoptotic pathway and is crucial for programmed cell death in response to damage. In skeletal muscle, caspase 8-mediated apoptosis plays a crucial role in eliminating damaged myonuclei and interstitial cells following intense or prolonged exercise, thereby supporting tissue remodeling and recovery [63]. However, excessive or dysregulated activation may delay regeneration and exacerbate muscle degradation, contributing to overuse injuries. Genetic variants within *CASP8*, such as rs3834129 ins/del and rs1045485 G/C, have been linked to musculoskeletal soft tissue injuries, including Achilles tendinopathy and noncontact ACL ruptures [64]. In addition, in a study conducted on 107 male athletes, Larruskain and coworkers [12] reported that the rs3834129 polymorphism was associated with the severity of muscle injury; specifically, carriers of the II and DD genotype showed more severe muscle injuries than heterozygotes. These findings suggest that *CASP8* polymorphisms may modulate individual susceptibility to exercise-induced muscle damage by influencing the balance between muscle cell death and regeneration.

### 2.6. Genes Associated with the Control of Vascular Tone and Muscle Perfusion

Genes involved in the regulation of vascular tone and muscle perfusion are essential for maintaining oxygen delivery, nutrient exchange, and the clearance of metabolic byproducts during and after exercise. Genetic variation within these pathways may influence vascular function, recovery efficiency, and overall susceptibility to muscle injury because impaired blood flow and microcirculatory dysfunction can delay repair processes and promote tissue vulnerability under repetitive or high-intensity loading [65]. The genes most frequently discussed in the context of cardiovascular regulation are *ACE*, *BDKRB2*, *AGT*, *HIF1A*, and *NOS3* [66].

The most studied gene associated with physical performance is *ACE*, which encodes angiotensin I-converting enzyme, a key element in the renin angiotensin system (RAS). This enzyme acts not only as an endocrine regulator but also within local cells and tissue, where it has diverse functions [67]. The I allele, which represents an insertion of 287 base pairs of Alu repeat elements in intron 16, is associated with lower circulating [68,69] and tissue ACE activity [66] and enhanced performance in endurance sports [67]. Moreover, the D allele, linked to deletion of the Alu sequence, is associated with increased enzyme activity [68] and improved power and strength performance [67]. This gene has also been described in the context of sports injuries. The common I/D genetic variant has been recently associated with susceptibility to inflammation and muscle damage after exercise; specifically, professional football players with the II genotype are more susceptible to developing muscle injuries [9,10]. Additionally, Yamin et al. [70] reported *ACE* genotype-dependent differences in circulating CK levels following eccentric exercise, indicating a potential protective role of the D allele against exercise-induced muscle damage. This observation was corroborated by Sierra et al. [11], who reported associations between *ACE* polymorphisms, inflammation, and muscle injury in Brazilian male marathon runners, with the DD genotype appearing to confer reduced susceptibility to muscle injury.

## 3. Candidate Genes Potentially Associated with Muscle Injury Risk in Athletes

Research on the genetic basis of muscle injury susceptibility is still at an early stage, with only a limited number of loci having been investigated. Current studies highlight the need for broader and more comprehensive studies that expand the most frequently examined variants to include additional genes and pathways potentially involved in muscle structure and function [26,34]. Many genes are candidates for future research, and the most promising genes are listed in Table 2. The selection of these candidate genes was based primarily on biological plausibility. Specifically, genes were included if their products play essential roles in muscle fiber structure, regeneration, extracellular matrix remodeling, energy metabolism, inflammation, or neuromuscular signaling. In addition, genes previously reported in the context of muscle-related disorders (e.g., muscular dystrophies, myopathies, or exercise-induced muscle damage) or showing differential expression following muscle injury or physical exercise were also considered. The biological functions of the products encoded by these genes indicate that changes in their sequences may play an important role in the development of an increased risk of muscle injury. Therefore, future studies should not only replicate previous findings to ensure their robustness across different populations and sports but also expand the analysis to include novel genetic markers with known or suspected roles in muscle tissue integrity, function, and ultimately musculoskeletal injury susceptibility.

Among the genes that should be investigated more extensively in future studies is titin (*TTN*). Titin is the third most abundant component in both skeletal and cardiac sarcomeres and extends from the Z-disk to the M-line. Titin filaments perform numerous functions in muscle contraction and force production, including structural and developmental roles in sarcomere organization, and mechanical roles in active and passive force regulation in muscles; they also serve as sensory and signaling mediators [71,72,73]. Previous studies have linked *TTN* variants to muscle properties such as stiffness, tension, strength, and flexibility, which are factors relevant to athletic performance and injury risk. The best-known *TTN* polymorphism, rs10497520 (C>T), results in the transformation of a lysine (Lys) residue into glutamic acid (Glu), which may favorably or unfavorably influence the properties of muscles and injury susceptibility. To date, this SNP has been linked to differences in passive stiffness, muscle fascicle length, athletic performance, and adaptation of skeletal and cardiac function to training [74,75,76,77,78], suggesting a possible role in muscle injury. However, studies conducted by Larruskain et al. [12] on another polymorphism in the *TTN* gene, rs2742327, did not reveal a significant association between genotype distribution and hamstring injuries in elite soccer players.

Another gene that seems to be important in the context of sport-related muscle injury is *MSTN*, which encodes myostatin. Myostatin acts as a negative regulator of skeletal muscle growth and development [79]. Reduced or absent myostatin activity may impair muscle quality, understood as the ability of the muscle to generate force relative to its size, which in turn could increase susceptibility to muscle injury. This effect is potentially mediated by disrupted mitochondrial biogenesis and/or altered protein turnover resulting from myostatin inhibition [80]. Given these physiological roles, SNPs within the *MSTN* gene are considered promising candidates for explaining interindividual variation in the risk of soft tissue injury. To date, only a few *MSTN* variants, such as K153R (rs1805086, 458 A>G), A55T (rs180565, 163 G>A), E164K (rs35781413, 490 G>A), P198A, I225T, rs11333758 (c.373 + 90delA), and c.373 + 5 G>A (rs397515373), have been studied in the context of sports genetics. *MSTN* polymorphisms are associated with differences in skeletal muscle mass, training-induced body changes, strength recovery following exercise-induced muscle damage, and elite athlete status [81,82,83,84]. Owing to the role of the gene product in the regulation of muscle growth and development, SNPs in the genes encoding *MSTN* and its receptors (activin a receptor type 2A and 2B—*ACVR2A* and *ACVR2B*) should be included in future studies.

Moreover, SNPs in the *RYR1* gene, which encodes the skeletal muscle ryanodine receptor responsible for calcium release during excitation–contraction coupling, have emerged as robust candidates for predicting susceptibility to exercise-induced muscle damage and injuries in athletes. Pathogenic and subclinical *RYR1* variants are well known to cause malignant hyperthermia (MH) and exertional rhabdomyolysis (ERM) by disrupting calcium homeostasis, leading to uncontrolled Ca^2+^ release, metabolic stress, and muscle breakdown [85]. Animal and clinical studies further demonstrate that even nonmanifesting carriers of *RYR1* variants may exhibit transient muscle stiffness, elevated CK levels, and reduced tolerance to exercise, which are factors directly relevant to sports performance and injury risk [86]. Given its well-described pathophysiology, extensive characterization in both clinical and exercise contexts, and readily available SNP data (e.g., rs63749869 and rs118192175) [87,88], *RYR1* stands out as an interesting genetic marker candidate.

Among the genes involved in inflammatory signaling pathways and cellular stress responses, the *TNF* gene is of particular interest because it is a potent proinflammatory cytokine that plays a central role in initiating and modulating the inflammatory cascade during muscle injury, especially in the context of eccentric exercise or overload. It influences satellite cell activation, muscle regeneration, and apoptosis, but excessive or prolonged TNF-α activity may delay recovery or exacerbate tissue degradation [89]. Several SNPs within the *TNF* promoter region, such as rs1800629 (−308 G>A), have been associated with altered cytokine expression levels and individual differences in postexercise inflammation and tissue repair capacity. Moreover, TNF-α interacts with other inflammatory mediators, such as IL-6 and IL-1β, creating a complex cytokine environment that can either facilitate recovery or exacerbate damage, depending on the individual’s genetic background [90].

Another promising gene associated with energy metabolism is *PPARGC1A*, which encodes the transcriptional coactivator PGC-1α, a master regulator of multiple metabolic pathways, with a direct impact on mitochondrial biogenesis, oxidation of fatty acids, metabolism of carbohydrates, thermogenesis, and reactive oxygen species detoxification [91,92,93]. Among the various genetic factors studied in relation to athletic performance, one of the most extensively examined within the *PPARGC1A* gene is the c.1444G>A (rs8192678) variant. This SNP leads to a glycine-to-serine substitution at amino acid position 482 of the PGC-1α protein (Gly482Ser), which may influence the protein’s regulatory activity. Evidence from several studies indicates that carriers of the A allele tend to exhibit reduced *PPARGC1A* expression, accompanied by lower aerobic performance and diminished metabolic efficiency during sustained exercise. Conversely, the G allele has been associated with enhanced gene expression and more effective endurance adaptations, suggesting a potential genetic advantage in endurance-oriented disciplines [94,95,96,97]. Although there is as yet no direct evidence linking rs8192678 variants with the incidence of muscle injury, the biological role of PGC-1α suggests that carriers of less favorable genotypes may be at increased risk of micro-damage and slower recovery under high training loads. Integrating *PPARGC1A* genotyping with physiological tests such as mitochondrial capacity and fatigue thresholds may thus support personalized injury-prevention and training strategies in athletes.

Recent advances in the field of psychogenetics, the study of how genetic variation influences psychological traits and behavior, suggest a novel dimension to athlete injury-risk profiling. For example, polymorphisms in genes such as dopamine receptor D2 (*DRD2*), catechol-O-methyltransferase (*COMT*), brain-derived neurotrophic factor (*BDNF*), and monoamine oxidase (*MAO*) have been associated with variations in conscientiousness and other personality traits in athletes, highlighting how gene–environment interactions may shape psychological profiles under high-demand athletic conditions [98]. Moreover, athletes experiencing musculoskeletal injuries are at elevated risk of psychological distress, and genetic variants regulating stress-response have been implicated in individual differences in resilience and recovery [99,100]. Taken together, these findings suggest that genetic markers of psychological regulation, stress resilience, and behavioral traits may interact with physical load, injury history, and recovery trajectories to modulate muscle-injury susceptibility in athletes. Incorporating psychogenetic profiling into longitudinal monitoring could therefore enrich personalized injury-prevention strategies by bridging physiological, psychological, and genetic risk domains.

Overall, the evidence indicates that the genes characterized in Table 2 represent a biologically plausible and underutilized set of candidates whose variants may significantly contribute to differences in muscle injury risk, recovery, and athletic performance. Incorporating them into future multigene panels or genome-wide analyses could provide crucial insights into the genetic network governing muscle resilience, recovery, and injury risk in athletes.

**Table 2:** Candidate genes potentially associated with muscle injury susceptibility for future investigation.

Gene *	Chromosomal Location *	Encoded Protein *	Protein Function *	Clinical Significance *	Possible Effect of Mutations on Muscle Injury	References
*ACTA1*	1q42.13	actin α 1	a crucial component of muscle fibers essential for muscle contraction and maintaining muscle structure	myopathies, including nemaline myopathy, congenital myopathy with excess of thin myofilaments, congenital myopathy with cores, and congenital myopathy with fiber-type disproportion	disrupts actin filament structure and muscle fiber integrity, leading to impaired contraction and increased muscle injury risk	[101]
*ANO5*	11p14.3	anoctamin 5	a multi-pass membrane enzyme catalyzes the hydrolysis of ATP coupled with the translocation of calcium from the cytosol to the sarcoplasmic reticulum lumen, and is involved in muscular excitation and contraction	muscle and bone disorders such as gnathodiaphyseal dysplasia	impairs membrane repair after microtears	[102]
*ATP2A1*	16p11.2	ATPase sarcoplasmic/endoplasmic reticulum Ca^2+^ transporting 1	an enzyme catalyzes the hydrolysis of ATP coupled with the translocation of calcium from the cytosol to the sarcoplasmic reticulum lumen; it is involved in muscular contraction and relaxation	Brody myopathy	defective muscle relaxation, reduced contractile efficiency, and increased susceptibility to exercise-induced muscle damage	[103]
*BGN*	Xq28	biglycan	a protein plays a role in the extracellular matrix of various tissues; it is involved in collagen fibril assembly, cell signaling, and may also regulate inflammation and innate immunity	X-linked spondyloepimetaphyseal dysplasia and thoracic aortic aneurysm syndrome	impairs collagen organization and weakened muscle–tendon integrity	[104]
*CACNA1S*	1q32.1	calcium voltage-gated channel subunit α1 S	it plays a critical role in muscle contraction by regulating calcium ion release within muscle cells	hypokalemic periodic paralysis, thyrotoxic periodic paralysis and malignant hyperthermia susceptibility	impairs edexcitation–contraction coupling, which may reduce contractile efficiency	[105]
*CAV3*	3p25.3	caveolin 3	scaffolding proteins for organizing and concentrating certain caveolin-interacting molecules	muscle disorders, including limb-girdle muscular dystrophy (LGMD) 1C, rippling muscle disease, and isolated hyperCKemia	membrane instability and impairs signal transduction	[106]
*CLCN1*	7q34	chloride voltage-gated channel 1	it regulates the electric excitability of the skeletal muscle membrane	recessive generalized myotonia congenita (Becker) and dominant myotonia (Thomsen)	membrane hyperexcitability and myotonia, which may increase mechanical stress on fibers	[107]
*GDF5*	20q11.22	growth differentiation factor 5	it regulates the development of numerous tissue and cell types, including cartilage, joints, brown fat, teeth, and the growth of neuronal axons and dendrites	acromesomelic dysplasia, brachydactyly, chondrodysplasia, multiple synostoses syndrome, proximal symphalangism, and susceptibility to osteoarthritis	alters tendon and muscle attachment sites	[108]
*DES*	2q35	desmin	forms a stable intracytoplasmic filamentous network connecting myofibrils to each other and to the plasma membrane	desmin-related myopathy, a familial cardiac and skeletal myopathy (CSM), and distal myopathies	muscle fiber disarray, weakness	[109]
*DMD*	Xp21.2-p21.1	dystrophin	forms a component of the dystrophin-glycoprotein complex (DGC), which bridges the inner cytoskeleton and the extracellular matrix	Duchenne muscular dystrophy, Becker muscular dystrophy, cardiomyopathy	impairs sarcolemma stability during contraction and increase susceptibility to muscle fiber damage compromise	[110]
*DYSF*	2p13.2	dysferlin	crucial to maintaining the structural integrity of muscle cells, involved in repairing damage to sarcolemma; also plays a role in muscle regeneration, vesicle trafficking, and calcium signaling	muscular dystrophies like Miyoshi myopathy and limb–girdle muscular dystrophy type 2B	defective membrane resealing after mechanical stress	[111]
*FBN2*	5q23.3	fibrillin-2	a component of connective tissue microfibrils and may be involved in elastic fiber assembly	congenital contractural arachnodactyly	weakening connective tissue support for muscle	[112]
*FGF9*	13q12.11	fibroblast growth factor 9	possesses broad mitogenic and cell survival activities, and is involved in a variety of biological processes, including embryonic development, cell growth, morphogenesis, tissue repair, tumor growth and invasion	multiple synostoses syndrome type 3, craniosynostosis and skeletal abnormalities	impairs muscle and skeletal development, regeneration, and vascularization	[113]
*FMOD*	1q32.1	fibromodulin	owing to the interaction with type I and type II collagen fibrils and in vitro inhibition of fibrillogenesis, the encoded protein may play a role in the assembly of extracellular matrix; it may also regulate TGF-beta activities by sequestering TGF-beta into the extracellular matrix.	osteoarthritis, tendon injuries, and certain fibrotic disorders	compromises ECM organization and tendon resilience	[114,115]
*FOXO3*	6q21	forkhead box O3	a trigger for apoptosis through expression of genes necessary for cell death	longevity, several cancer risk, neurodegeneration, and cardiometabolic disorders	enhance proteolysis and reduce regenerative capacity	[116]
*HSPG2*	1p36.12	heparan sulfate proteoglycan 2	a versatile protein with critical roles in maintaining tissue structure, regulating cell behavior, and influencing vascular function	Schwartz–Jampel syndrome type 1, Silverman-Handmaker type of dyssegmental dysplasia, and tardive dyskinesia	impair structural support, neuromuscular junction stability, and tissue repair	[117]
*IGF1*	12q23.2	Insulin-like growth factor 1	a versatile hormone that is essential for growth, development, and metabolic regulation	insulin-like growth factor I deficiency	reduces muscle growth, regeneration, and repair capacity	[118]
*ITGB2*	21q22.3	integrin subunit β 2	integrin is vital for cell adhesion and signaling, particularly in the immune system	Leukocyte Adhesion Deficiency type 1	disrupt leukocyte adhesion and immune signaling, which may impair inflammatory response and tissue repair	[119]
*LMNA*	1q22	lamin A/C	structural protein involved in nuclear stability, chromatin structure and gene expression	Emery–Dreifuss muscular dystrophy, familial partial lipodystrophy, limb girdle muscular dystrophy, dilated cardiomyopathy, Charcot–Marie–Tooth disease, and Hutchinson–Gilford progeria syndrome	impairs nuclear integrity, alters mechanotransduction and defective muscle regeneration	[120]
*NEB*	2q23.3	nebulin	component of the cytoskeletal matrix that coexists with the thick and thin filaments within the sarcomeres of skeletal muscle	nemaline myopathy	impair force generation and greater susceptibility to muscle fiber damage	[121]
*MTM1*	Xq28	myotubularin 1	enzyme plays a crucial role in muscle cell development and maintenance by acting as a phosphatase	X-linked myotubular myopathy	defective excitation–contraction coupling	[122]
*MSTN*	2q32.2	myostatin	negative regulator of skeletal muscle cell proliferation and differentiation	increased skeletal muscle mass in humans and other mammals	alters muscle fiber composition and elasticity	[79,80,81,82,83,84]
*MYH1-4* *MYH6-7*	17p13.1 14q11.2	myosin heavy chain 1–7	a key component of fast-twitch muscle fibers and is involved in skeletal muscle contraction	MYH1—autoimmune/inflammatory myopathies; MYH2—congenital myopathy with ophthalmoplegia; MYH3 → distal arthrogryposis syndromes; MYH7—cardiomyopathies and skeletal myopathies (Laing distal, myosin storage)	disrupt sarcomeric function, fiber type specialization, and contractile force generation	[123]
*MYOD1*	11p15.1	myogenic differentiation 1	a protein that acts as a transcription factor, specifically activating the expression of genes necessary for muscle cell differentiation, the formation of muscle fibers, and muscle regeneration	Rhabdomyosarcoma and other cancers	impairs muscle regeneration and repair after damage	[124]
*MYOG*	1q32.1	myogenin	transcription factor that can induce myogenesis in a variety of cell types in tissue culture; it is essential for the development of functional skeletal muscle	implicated in congenital muscle hypoplasia (experimental evidence) and serves as a hallmark of rhabdomyosarcoma in diagnostics	impair regeneration and repair of damaged fibers	[125]
*MYF5*	12q21.31	myogenic factor 5	transcription factor involved in several processes, including muscle cell fate commitment, positive regulation of cell differentiation, and skeletal muscle cell differentiation	potential contributors to congenital myopathies, and its expression serves as a marker in rhabdomyosarcoma	reduce regenerative capacity after damage	[126]
*MYF6*	12q21.31	myogenic factor 6	a transcription factor involved in muscle development and regeneration	congenital myopathy, facioscapulohumeral muscular dystrophy, and rhabdomyosarcoma	impair regeneration and repair	[126]
*PAX3*	2q36.1	paired box 3	a transcription factor crucial for embryonic development, particularly in the formation of neural crest cells and skeletal muscle	Waardenburg syndrome, craniofacial-deafness-hand syndrome, and alveolar rhabdomyosarcoma	impairs muscle regeneration after damage	[127,128]
*PAX7*	1p36.13	paired box 7	a transcription factor crucial for the development and maintenance of skeletal muscle and the nervous system, it plays a key role in the regulation of muscle satellite cells, ensuring their self-renewal and differentiation into muscle fibers	rhabdomyosarcoma	impairs muscle regeneration after damage	[129,130]
*PPARGC1A*	4p15.2	PPARG coactivator 1 α	a transcriptional coactivator of multiple metabolic pathways, with a direct impact on mitochondrial biogenesis, oxidation of fatty acids, metabolism of carbohydrates, thermogenesis, and reactive oxygen species detoxification	metabolic, cardiovascular, neurodegenerative, and muscle-related disorders	impairs mitochondrial biogenesis and oxidative metabolism, reducing fatigue resistance and recovery	[91,92,93,94,95,96,97]
*RYR1*	19q13.2	ryanodine receptor 1	functions as a calcium release channel in the sarcoplasmic reticulum but also serves to connect the sarcoplasmic reticulum and transverse tubule	malignant hyperthermia susceptibility, central core disease, and minicore myopathy with external ophthalmoplegia	dysregulates excitation–contraction coupling, abnormal calcium homeostasis, and increases susceptibility to exertional rhabdomyolysis	[85,86,87,88]
*SCN4A*	17q23.3	sodium voltage-gated channel α subunit 4	responsible for the generation and propagation of action potentials in muscle	several myotonia and periodic paralysis disorders	alters excitability and myotonic or periodic paralysis phenotypes, which may increase mechanical stress on fibers	[131,132]
*SGCA SGCB SGCD SGCG*	17q21.334q12 5q33.2 13q12.12	Sarcoglycan α, β, δ, γ	transmembrane components in the dystrophin–glycoprotein complex which help stabilize the muscle fiber membranes and link the muscle cytoskeleton to the extracellular matrix	limb–girdle muscular dystrophy	weakens sarcolemmal stability during contraction and muscle fiber degeneration	[133]
*TGFBI*	5q31.1	transforming growth factor β	protein plays a role in cell–collagen interactions	multiple types of corneal dystrophy	impairs tissue repair processes	[134,135]
*TGFBR3*	1p22.1	transforming growth factor β receptor 3	receptor is a membrane proteoglycan that often functions as a co-receptor with other TGF-beta receptor superfamily members influencing cell growth, differentiation, and extracellular matrix remodeling	various cancers	impairs ECM homeostasis, inflammation control, and tissue repair	[136]
*TNF*	6p21.33	tumor necrosis factor	regulation of a wide spectrum of biological processes including cell proliferation, differentiation, apoptosis, lipid metabolism, and coagulation	cerebral malaria, septic shock, and Alzheimer disease	exacerbates muscle fiber damage and delay repair	[89,90]
*TTN*	2q31.2	titin	a crucial component of muscle cells, particularly in the heart and skeletal muscles; titin acts as a biological spring within structures called sarcomeres, the basic units of muscle contraction, and is essential for muscle structure, flexibility, stability, and the ability to stretch; it also plays a role in chemical signaling and sarcomere assembly	Dilated Cardiom Hypertrophic Cardiomyopathy yopathy, skeletal muscle diseases, including tibial muscular dystrophy, limb–girdle muscular dystrophy, and centronuclear myopathy	increases muscle stiffness, worsens force transmission and repair capacity	[71,72,73,74,75,76,77,78]
*TPM2*	9p13.3	tropomyosin 2	crucial for muscle contraction; it plays a key role in regulating the interaction between actin and myosin filaments, the proteins responsible for generating the force needed for muscle movement	cap disease, nemaline myopathy and distal arthrogryposis syndromes	impairs actin–myosin interaction and force generation	[137,138]
*TPM3*	1q21.3	tropomyosin 3	provides stability to actin filaments and regulates access of other actin-binding proteins	Myopathies, including nemaline myopathy, congenital fiber-type disproportion, cap myopathy, and neuromuscular transmission defects	reduces contractile efficiency and structural stability	[137,138,139]
*VEGFA*	6p21.1	vascular endothelial growth factor A	growth factor induces proliferation and migration of vascular endothelial cells	pathological angiogenesis (eye disease, cancer) and vascular dysfunction (cardiovascular, inflammatory disorders)	impair angiogenesis and muscle perfusion, limiting oxygen and nutrient delivery during recovery	[140]

* Data form National Center for Biotechnology Information (NCBI).

## 4. Perspectives

To date, most genetic research on injury susceptibility in athletes has focused on tendons and ligaments, with particular attention given to genes that influence collagen structure and connective tissue integrity [7]. In contrast, the genetic basis of muscle injuries, such as strains, tears, and exercise-induced muscle damage, has received relatively little attention. Despite the high prevalence and performance impact of muscle injuries in many sports [1], they remain underrepresented in genomic studies. This imbalance highlights a clear gap in the literature. Future research should aim to systematically investigate genetic variants associated with muscle-specific injury risk, recovery, and regeneration using candidate gene approaches, genome-wide association studies (GWASs), and next-generation sequencing (NGS) technologies. A better understanding of the molecular underpinnings of muscle injury susceptibility could contribute to more personalized prevention strategies and improved athlete care.

The large majority of published studies have followed a single or a small number of SNPs using case–control study designs, which did not provide comprehensive conclusions about the combined impact of these SNPs on the susceptibility to injuries. Therefore, simultaneously analyzing numerous polymorphic sites is more advantageous and may offer additional insights into the genetic predisposition to the occurrence or absence of injuries. One simple approach is to increase the number of variants analyzed and calculate the total genotype score (TGS), which reflects the additive effect of genotypes on the prediction of complex traits such as the development of disease, athletic performance or injury risk [13,141,142,143]. TGS modeling allows, among other methods, the differentiation of a group of professional athletes from nonathletes and the characterization of athletes with different susceptibilities to sport-related injuries or training-induced body changes [36,93]. However, owing to the need to simultaneously analyze large numbers of SNPs, to date, few authors have decided to use this valuable method. For example, the latest study conducted by Massidda et al. [13] on a group of 64 Italian male top-level football players revealed that the combination of the *ACE* rs4341, *ACTN3* rs1815739, *COL5A1* rs2722, and *MCT1* rs1049434 gene variants influences muscle injury risk. These results demonstrated a statistically significant association between the TGS values of injured and noninjured athletes, with a TGS cut-off point of 56.2 a.u. to discriminate these two groups. Compared with players with lower TGSs, football players with a TGS above this value had an injury odds ratio of 3.5; however, the severity of injury does not seem to be affected by the SNPs included in the model [13]. Future studies should involve more genetic markers and a greater number of participants.

The use of NGS technologies in the study of genetic predisposition to muscle injuries offers several important advantages over traditional genotyping approaches. Unlike candidate gene studies, which are limited to preselected variants, sequencing enables comprehensive analysis of entire genes, exomes, or genomes, allowing the identification of both common and rare variants potentially associated with injury susceptibility [144]. This analysis is particularly relevant for complex traits such as muscle damage, which may be influenced by a wide range of genetic factors that act in combination. Sequencing also allows researchers to discover novel variants, including those in regulatory or noncoding regions, that may affect gene expression and tissue-specific function. Additionally, whole-gene or whole-exome sequencing can reveal compound heterozygosity or gene–gene interactions that may be missed with more targeted approaches. Applying sequencing in diverse athletic populations could improve the detection of population-specific or sport-specific genetic risk profiles, contributing to a more nuanced understanding of muscle injury mechanisms. Ultimately, integrating sequencing data with phenotypic, biomechanical, and transcriptomic information may pave the way for personalized injury prevention strategies and genetically informed training programs [145,146].

To complement genomic approaches, epigenetic studies, such as those exploring DNA methylation, histone modifications, and noncoding RNA regulation [147], should also be integrated into future research. Epigenetic mechanisms can modulate gene expression in response to training load, muscle damage, inflammation, or recovery protocols and may help explain the variability in injury susceptibility and healing outcomes that cannot be attributed to DNA sequence alone. For instance, changes in DNA methylation patterns in genes involved in inflammation, muscle repair, or fibrosis have been observed following intense physical activity and may differ between individuals [100,148]. The incorporation of epigenetic profiling alongside genomic data offers a more dynamic and comprehensive view of the biological processes involved in muscle injury and recovery and could lead to precision medicine approaches tailored to an athlete’s genetic and epigenetic landscape.

Emerging genome-editing tools such as CRISPR/Cas9 offer novel avenues to validate the functional impact of gene variants implicated in muscle injury risk and to refine our interpretation of candidate polymorphisms. For example, a recent review highlights how CRISPR/Cas9 has been successfully applied to correct mutations in neuromuscular disorders, demonstrating proof of concept in skeletal muscle tissue models [149,150,151,152]. While these applications focus mainly on disease states (e.g., dystrophin correction in muscle dystrophy) [150,151], the methodology points to a potential future in sports-genetics research: editing or modeling variants in muscle-repair, regeneration and extracellular matrix pathways could clarify causative mechanisms and distinguish benign associations from true injury-predisposing alleles. Incorporating such evidence-based gene-editing research thus strengthens the scientific validity of candidate gene selection and helps prevent contradictory interpretations of polymorphisms when applied to athlete injury risk.

The risk of muscle injury is influenced not only by genetic factors but also by their interaction with environmental variables. Gene–environment interactions play a crucial role in modulating individual susceptibility to injury, where specific genotypes may alter the body’s response to factors such as training load, recovery protocols, biomechanical stress, and nutrition. For instance, individuals carrying certain polymorphisms may exhibit reduced tissue resilience or impaired repair mechanisms under excessive or poorly managed physical stress [153]. Therefore, future research should move beyond isolated genetic analysis and adopt an integrative approach that combines genetic data with detailed environmental, physiological, and lifestyle variables. Such studies could lead to more accurate risk assessment and the development of personalized training and injury prevention strategies in physically active populations.

## 5. Conclusions

In the future, the outcome of molecular research will be the development of a foundation for a prevention program that targets musculoskeletal injuries in physically active individuals. A key objective of this program will be the translation of scientific findings into practical preventive strategies, which will be highly important for the advancement of personalized medicine and physical culture sciences. The ultimate beneficiaries of this initiative will include all individuals engaging in physical activity, both amateur and professional, as well as instructors, personal trainers, coaches across various sports disciplines, physiotherapists, and orthopedic physicians. Knowledge of the risk of specific types of soft tissue injuries will enable the individualized management of athletes via appropriately tailored training, load regulation, and the application of other protective measures to better safeguard those with genetic predispositions. Even for physically active individuals who are not professional athletes, genetic insights may prove useful in reducing injury risk via targeted strengthening exercises and improved neuromuscular control. This achievement broadens our understanding of the genetic basis of physical activity and health and has potentially significant implications for sports medicine and individual and population-level health strategies. Therefore, continuing research aimed at identifying the genetic determinants of sport-related muscle injuries is essential.

## Figures and Tables

**Table 1:** Genetic markers significantly associated with susceptibility to sport-related muscle injuries.

Gene *	Chromosomal Location *	Encoded Protein *	Polymorphism *	Polymorphism Location (GRCh38) *	(1) Muscle Injury Risk, (2) Severity, (3) Incidence or (4) Recovery Time	References
Regulators of vascular function and perfusion						
*ACE*	17q23.3	angiotensin I-converting enzyme	rs4646994 or rs4341, I/D	intron 16	(1) ↑ I, ↓ D	[9,10]
*AGT*	1q42.2	angiotensinogen	rs699, A/G, Met235Thr	1:230,710,048	(1) ↑ Met, ↓ Thr	[11]
*BDKRB2*	14q32.2	bradykinin receptor B2	rs5810761, +9/−9	exon 1	(1) ↑ −9, ↓ +9	[11]
*HIF1A*	14q23.2	Hypoxia-inducible factor 1α	rs11549465, C/T, Pro582Ser	exon 12 14:61,740,839	(1) ↑ CC, ↓ CT	[12]
*NOS3*	7q36.1	nitric oxide synthase 3	rs1799983, G/T, Glu298Asp	exon 7 7:150,999,023	(3) ↑ G, ↓ T	[12]
Genes involved in the structural organization of muscle fibers and ECM						
*ACTN3*	11q13.2	actinin alpha 3	rs1815739, C/T, R577X	exon 16 11:66,560,624	(1), (2), (3), (4) ↑ X, ↓ R	[10,13,14,15,16,17,18,19]
*ADAMTS14*	10q22.1	ADAM metallopeptidase with thrombospondin type 1 motif 14	rs4747096, A/G, Glu/Gln	exon 21 10:70,758,253	(2) ↑ AG, ↓ AA	[12]
*COL1A1*	17q21.33	collagen type I alpha 1 chain	rs1107946, A/C	promoter 17:50,203,629	(1) ↑ AA, ↓ CC, AC	[20]
*COL5A1*	9q34.3	collagen type V alpha 1 chain	rs12722, C/T	3′ UTR 9:134,842,570	(2) ↑ CT, ↓ CC, TT	[21,22]
					(1) ↓ TT	[23]
			rs16399, I/D	3′ UTR 9:134,843,389	(3) ↑ DI, ↓ DD, II	[12]
*COL22A1*	8q24.23-q24.3	collagen type XXII α-1 chain	rs11784270, A/C	intron 1 8:138,908,388	(1) ↑ A, ↓ C	[24]
			rs6577958, T/C	intron 1 8:138,903,960	(1) ↑ T, ↓ C	
*DCN*	12q21.33	decorin	rs516115, A/G	intron 3 12:91,163,515	(3) ↑ A, ↓ G	[12]
*ELN*	7q11.23	elastin	6124052A>G (no rs number)	unknown	(2) ↑ AA, ↓ AG, GG	[25]
*MLCK*	3q21.1	myosin light chain kinase	rs28497577, C/A,	5′ UTR 3:123,793,780	(1) ↑ C, ↓ A	[26]
			rs2700352, C/T	5′ UTR 3:123,831,616	(2) ↑ TT, ↓ CT, CC	[12]
*MMP1*	11q22.2	matrix metalloproteinase 1	rs1799750	promoter	(3) ↑ DD, DI, ↓ II	[12]
*MMP3*	11q22.2	matrix metalloproteinase 3	rs679620, G/A, Glu45Lys	exon 2 11:102,842,889	(1) ↑ A, ↓ G	[12]
*MMP12*	11q22.2	matrix metalloproteinase 12	rs2276109, A/G	promoter	(3) ↑ A, ↓ G	[12]
*TNC*	9q33.1	tenascin C	rs2104772, A/T	exon 17 9:115,046,506	(3) ↑ A, ↓ T	[12]
*VWA5A* interaction region	11q24.2	von Willebrand factor A domain containing 5A	rs12807854 T/C	intergenic region 11:124,066,891	(1) ↑ C, ↓ T	[27]
Genes associated with metabolism and energy homeostasis						
*AMPD1*	1p13.2	adenosine monophosphate deaminase 1	rs17602729, C/T, Gln12Stop	exon 2 1:114,693,436	(1) ↑ T, ↓ C	[26]
*CKM*	19q13.32	creatine kinase, M-type	rs8111989, A/G	3′ UTR 19:45,305,950	(2) ↑ AG, ↓ GG	[28]
*MCT1*	1p13.2	monocarboxylate transporter 1	rs1049434, A/T, Glu490Asp	exon 5 1:112,913,924	(3) ↑ A, ↓ T	[13,29]
Genes associated with membrane integrity, ion transport, and signaling pathways						
*CASP8*	2q33.1	caspase 8	rs3834129, I/D	promoter 2:201,232,809	(2) ↑ DD, II, ↓ ID	[12]
*GEFT*	12q13.3	Rho guanine nucleotide exchange factor	rs11613457, A/G	cytogenetic region 12:57,618,450	(4) ↑ AG, ↓ GG	[22]
Genes related to muscle growth, development, and regeneration						
*IGF2*	11p15.5	insulin like growth factor 2	rs3213221, C/G	intron 1 11:2,135,814	(2) ↑ GG, CC, ↓ GC	[21,22,25]
*ESR1*	6q25.1-q25.2	estrogen receptor 1	rs2234693, C/T	intron 1 6:151,842,200	(1) ↑ T, ↓ C	[30]
*HGF*	7q21.11	hepatocyte growth factor	rs5745678, C/T	3′ UTR 7:81,742,731	(2, 4) ↑ C, ↓ T	[22]
			rs5745697, A/C	intron 7:81,728,033	(3) ↑AA, AC, ↓CC (2), (4) ↑CC, ↓ AA, AC	
			rs1011694, A/T	intron 7:81,703,677	(3) ↑ TT, TA, ↓ AA (2) ↑ AA ↓ TT, TA,	
*LIF*	22q12.2	leukemia inhibitory factor	rs929071, C/T	intron 20:42,350,790	(4) ↑ CT, CC, ↓ TT	[22]
*SOX15*	17p13.1	SRY-box transcription factor 15	rs4227, G/T	3′ UTR 17:7,587,859	(3) ↑ G, ↓ T	[22]
*VDR*	12q13.11	vitamin D receptor	rs7975232, ApaI, C/A	intron 8 12:47,845,054	(1) ↑ GG	[31,32]
Genes involved in inflammatory and stress-response pathways						
*IL1A*	2q14.1	interleukin 1 α	rs1800587, C/T	promoter 2:112,785,383	(2) ↑ CT, ↓ CC, TT	[12]
*IL6*	7p15.3	interleukin 6	rs1800795, G/C	promoter 7:22,727,026	(1) ↑ GG, ↓ GC, CC	[12]
					(1) ↑ CC, ↓ GC, GG	[33]
*CCL2*	17q12	C-C motif chemokine ligand 2	rs2857656, G/C	promoter 17:34,254,988	(2) ↑ GG, ↓ CC, CG	[21,22]

* Data form National Center for Biotechnology Information (NCBI). Abbreviations: I—insertion; D—deletion; Met—methionine; Thr—threonine; C—cytosine; T—thymine; G—guanine; A—adenine; X—stop codon at position 577 of ACTN3; R—codon for arginine at position 577 of ACTN3.

## Data Availability

No new data were created or analyzed in this study. Data sharing is not applicable to this article.

## Abbreviations

A	Adenine
ACE	Angiotensin I-converting enzyme
ACL	Anterior cruciate ligament
ACTA1	Actin α 1
ACTN3	Actinin alpha 3
ACVR2A	Activin a receptor type 2A
ACVR2B	Activin a receptor type 2B
ADAMTS14	A disintegrin and metalloproteinase with thrombospondin motifs 14
AGT	Angiotensinogen
AMPD1	Adenosine monophosphate deaminase 1
ANO5	Anoctamin 5
ATP	Adenosine triphosphate
ATP2A1	ATPase sarcoplasmic/endoplasmic reticulum Ca^2+^ transporting 1
BDKRB2	Bradykinin receptor B2
BDNF	Brain-derived neurotrophic factor
BGN	Biglycan
C	Cytosine
CACNA1S	Calcium voltage-gated channel subunit α1 S
CASP8	Caspase 8
CAV3	Caveolin 3
CCL2	C-C motif chemokine ligand 2
CK	Creatine kinase
CKM	Creatine kinase, M-type
CLCN1	Chloride voltage-gated channel 1
COL1A1	Collagen type I alpha 1 chain
COL5A1	Collagen type V alpha 1 chain
COL22A1	Collagen type XXII α-1 chain
COMT	Catechol-O-methyltransferase
DCN	Decorin
D	Deletion
DES	Desmin
DMD	Dystrophin
DRD2	Dopamine receptor D2
DYSF	Dysferlin
ECM	Extracellular matrix
ELN	Elastin
eNOS	Endothelial nitric oxide synthase
ERM	Exertional rhabdomyolysis
ESR1	Estrogen receptor 1
FBN2	Fibrillin-2
FGF	Fibroblastic growth factor
FGF9	Fibroblastic growth factor 9
FMOD	Fibromodulin
FOXO3	Forkhead box O3
G	Guanine
GDF5	Growth differentiation factor 5
GEFT	Rho guanine nucleotide exchange factor
GWAS	Genome-wide association studies
HGF	Hepatocyte growth factor
HIF1A	Hypoxia inducible factor 1α
HSPG2	Heparan sulfate proteoglycan 2
I	Insertion
IGF1	Insulin-like growth factor 1
IGF2	Insulin-like growth factor 2
IL1A	Interleukin 1 α
IL1B	Interleukin 1 β
IL6	Interleukin 6
ITGB2	Integrin subunit β 2
LIF	Leukemia inhibitory factor
LMNA	Lamin A/C
MAO	Monoamine oxidase
MCT1	Monocarboxylate transporter 1
MH	Malignant hyperthermia
Met	Methionine
MLCK	Myosin light chain kinase
MMP1	Matrix metalloproteinase 1
MMP3	Matrix metalloproteinase 3
MMP12	Matrix metalloproteinase 12
MRF6	Myogenic factor 6
MSTN	Myostatin
MTM1	Myotubularin 1
MYF5	Myogenic factor 5
MYH1-7	Myosin heavy chain 1–7
MYOD1	Myogenic differentiation 1
MYOG	Myogenin
NCBI	National Center for Biotechnology Information
NEB	Nebulin
NGS	Next-generation sequencing
NO	Nitric oxide
NOS3	Nitric oxide synthase 3
PAX3	Paired box 3
PAX7	Paired box 7
PPARGC1A	Peroxisome proliferator-activated receptor gamma coactivator 1 α
R	Codon for arginine at position 577 of ACTN3
RYR1	Ryanodine receptor 1
SCN4A	Sodium voltage-gated channel α subunit 4
SGCA	Sarcoglycan α
SGCB	Sarcoglycan β
SGCD	Sarcoglycan δ
SGCG	Sarcoglycan γ
SNP	Single nucleotide polymorphism
SOX15	Sex-determining region Y (SRY)-box transcription factor 15
T	Thymine
TGFBI	Transforming growth factor β
TGFBR3	Transforming growth factor β receptor 3
TGS	Total genotype score
Thr	Threonine
TNC	Tenascin C
TNFβ	Transforming growth factor-β
TPM2	Tropomyosin 2
TPM3	Tropomyosin 3
TTN	Titin
VEGFA	Vascular endothelial growth factor A
VDR	Vitamin D receptor
VWA5A	Von Willebrand factor A domain containing 5A
X	Stop codon at position 577 of ACTN3
